# Four new species of *Russula* subsection *Roseinae* from tropical montane forests in western Panama

**DOI:** 10.1371/journal.pone.0257616

**Published:** 2021-10-13

**Authors:** Cathrin Manz, Slavomír Adamčík, Brian P. Looney, Adriana Corrales, Clark Ovrebo, Katarína Adamčíková, Tina A. Hofmann, Felix Hampe, Meike Piepenbring

**Affiliations:** 1 Mycology Research Group, Faculty of Biological Sciences, Goethe University Frankfurt am Main, Frankfurtam Main, Germany; 2 Plant Science and Biodiversity Center, Slovak Academy of Sciences, Bratislava, Slovakia; 3 Department of Biology, Clark University, Worcester, MA, United States of America; 4 Centro de Investigaciones en Microbiología y Biotecnología-UR (CIMBIUR), Facultad de Ciencias Naturales, Universidad del Rosario, Bogotá, Colombia; 5 Department of Biology, University of Central Oklahoma, Edmond, OK, United States of America; 6 Department of Plant Pathology and Mycology, Institute of Forest Ecology, Slovak Academy of Sciences Zvolen, Nitra, Slovakia; 7 Mycological Research Center (CIMi), Herbarium UCH, Autonomous University of Chiriquí (UNACHI), David, Chiriquí Province, Panama; 8 Wetzlarer Str. 1, Butzbach, Germany; Friedrich Schiller University, GERMANY

## Abstract

Species of the genus *Russula* are key components of ectomycorrhizal ecosystems worldwide. Nevertheless, their diversity in the tropics is still poorly known. This study aims to contribute to the knowledge of the diversity of *Russula* species classified in subsection *Roseinae* based on specimens recently collected in tropical montane rainforests in western Panama. A five gene multilocus phylogeny based on the nuclear markers ITS nrDNA, *MCM7*, *RPB1*, *RPB2* and *TEF-1α* was constructed to identify the systematic position of 22 collections from Panama. Four new species, *Russula cornicolor*, *Russula cynorhodon*, *Russula oreomunneae* and *Russula zephyrovelutipes* are formally described and illustrated. None of the four species are sister species and they are more closely related to North American or Asian species. Two of the newly described species were associated with the ectomycorrhizal tree species *Oreomunnea mexicana*, while the other two species were associated with *Quercus* species. All four species are so far only known from mountains in western Panama.

## Introduction

Exploring fungal diversity in the tropics is a challenging and urgent task. Even ectomycorrhizal (ECM) fungi with large conspicuous fruiting bodies are underexplored due to a low number of mycologists, only sporadic development of fruiting bodies, often difficult access to their habitats, weather conditions favouring a rapid decay of fungal tissues, bureaucratic barriers, and a lack of funding [1]. As a result, our understanding of ECM fungi and their ecological role in the neotropics is still in its infancy [2, 3]. DNA metabarcoding of soil samples reveals a relatively low number of ECM fungal species in central America [4] emphasizing the need for focused studies of habitats with dominant stands of ECM host tree species.

The hyper-diverse ECM genus *Russula* Pers. is a taxonomically challenging genus because experience and detailed morphological observations are needed to describe and define species delimitations. With about 1.300 accepted and more than 2.000 estimated species, it is the second largest genus of ECM fungi worldwide after *Cortinarius* [5–7].

Approximately 3100 species of fungi are known from Panama [8, 9]. Among these species, there are nine species of *Russula* recorded for the entire country [6, 10–12]. In contrast to this, a high number of 31 OTUs of *Russula* spp. were detected from root tips of a single tree species in the Fortuna Forest Reserve in western Panama [13]. However, without reference sequences obtained from correctly identified fruiting bodies of described species, OTUs cannot be assigned species designations [14]. Additionally, the observation of fruiting bodies is necessary as proof of metabolic activity of a given fungus in its habitat. Environmental DNA sequencing can lead to false ecological conclusions, e. g., when DNA of resting spores or hyphal fragments is detected.

Extant species of *Russula* subsection *Roseinae* Sarnari diverged about three to one million years ago [15]. They are members of the most diverse and recently derived major lineage in the genus, the Crown clade, and are currently placed in the subgenus *Russula* Buyck & V. Hofst. [16, 17]. Fourteen species are classified in subsect. *Roseinae* and morphologically characterized by a pink, red or whitish pileus; a white to pale cream spore print; predominantly mild taste; context and lamellae turning eosin red with sulfo vanillin; absence of pileocystidia; and presence of primordial hyphae with acid resistant incrustations in the pileipellis on top of a pseudoparenchymatic layer [18]. By this distinctive morphology species of subsect. *Roseinae* are easily recognized among the many red-capped *Russula* species.

In Europe, *Russula* subsect. *Roseinae* is represented by two widely accepted species, *Russula velutipes* Velen. and *Russula minutula* Velen. [19]. In North America, *Russula albida* Peck and *Russula peckii* Singer are well known *Roseinae* members. In the phylogenetic study of species classified as *Roseinae* in the Eastern USA recently published by Looney et al. [20] seven species of this subsection are recognised. *Russula nigrescentipes* Peck that was formerly classified in subsect. *Roseinae* [18] was excluded. *Russula rimosa* Murrill is only known from its type collection from the USA and is classified in subsect. *Roseinae* based on its morphology only [18], because any attempt of sequencing DNA of the species have been unsuccessful. Two species of subsect. *Roseinae* from China and two further species from India indicate a circumpolar distribution in the Northern Hemisphere for this systematic relationship [21–23]. In total thirteen described species in subsect. *Roseinae* are known up to know.

The subsect. *Roseinae* is currently one of the most intensively studied lineages in the genus at a taxonomic level [20]. Therefore, it represents an ideal target for the discovery of new species in underexplored areas. Based on morphology and preliminary analyses of barcode sequences of the ITS rDNA region of more than 300 collections of *Russula* spp. made by authors of this study, we chose 22 collections belonging to subsect. *Roseinae* recently collected in tropical montane forests of the Chiriquí province in Western Panama. The aim of this study is to identify the taxonomic status and phylogenetic position of species represented by these collections. The species are based on multilocus phylogenetic analyses and formally described with detailed macro- and micromorphological data following the standards established by Adamčík et al. [6].

## Material and methods

### Sampling

The fruiting bodies examined in this study were collected during field trips to the Chiriquí region in Western Panama in 2007, 2012–2014 and 2018. The material was air dried with a dehydrator at 40°C. Dried collections are deposited at the herbaria of the Botanische Staatssammlung München (M), of the Autonomous University of Chiriquí (UCH), at the University of Arizona (ARIZ) and at the Florida Museum of Natural History (FLAS). Additionally, the holotype collections of the European taxa *Russula aurora* f. *armeniaca* Reumaux, *Russula aurora* var. *gemella* Reumaux & Frund, *Russula roseoalbescens* Reumaux & Frund and *Russula spurcata* Reumaux & Frund were analysed.

### Morphological analysis

Most fruiting bodies were described and photographed in fresh condition with colour designations according to Kornerup and Wanscher [24]. The colour reactions of stipe and gill surfaces after the application of FeSO_4_, Guaiac-solution and sulfo vanillin were tested. Microscopic observations followed the standards proposed by Adamčík et al. [6]. Drawings were prepared using a drawing attachment (U-DA) mounted on an Olympus CX41 microscope at a projection scale of x2000. Preparation of samples for Scanning electron microscopy (SEM) was carried out as described in Koch et al. [25]. SEM Photographs were taken with a Hitachi S-530 microscope with an applied voltage of 20–25 kV and a magnification of up to 8.000 times. The morphological observations from our own studied material were compared to the morphological data of North American species of subsection *Roseinae* published by Looney et al. [20]. For each species described in this study three to five recent collections were analysed size of microscopic strures were estimated based on 20 to 30 measurements per collection.

### Molecular genetic analysis

Genomic DNA was extracted from fragments of fresh material preserved in CTAB (Cetyl trimethylammonium bromide) buffer using the CTAB extraction method described in Nuytinck and Verbeken [26]. The innuPREP Plant DNA Kit (analytikjena, Jena, Germany) was used for DNA extraction from dry material following the manufacturer’s instructions.

Five nuclear markers used in a previous study on *Russula* subsect. *Roseinae* [15] were amplified and sequenced: (1) ITSnrDNA, using primers ITS-1F and ITS4 [27, 28], (2) *MCM7*, using primers *MCM7*-709F and *MCM7*-1348R [29], (3) *RPB1*, using primers gAf [30] and fCr [31], (4) *RPB2* using primers b6F and 7.1R [32], and (5) *TEF-1α*, using primers EF1-983F and EF1-2218R [33]. Some cases required to use primers *RPB1*-F3 and *RPB1*-R4 to amplify partial sequences of *RPB1* [34]. Forward and reverse sequences were assembled into contigs and edited with Geneious Prime 2020.2 (Biomatters limited, Auckland, New Zealand). The newly generated sequences were aligned together with a multi-locus dataset of worldwide *Roseinae* samples retrieved from Looney et al. [20] and aligned using MAFFT version 7 [35] with default settings. The resulting alignment was edited with Geneious Prime 2020.2. A strongly variable coding region of *RPB2* composed of mostly repeating codons of variable length as well as the gene coding region of the ribosomal small subunit 5.8S were excluded from the analysis. The alignment was partitioned into ITS, *MCM7*, *RPB1* intron and exon, *RPB2* and *TEF-1α* intron and exon. Maximum likelihood (ML) and Bayesian analyses were conducted on the CIPRES Science Gateway [36]. An initial multi-locus constraint tree was inferred for samples with at least one locus in addition to the ITS. ML analysis was conducted using RaxML-HPC2 on XSEDE version 8.0.24 [37], with the Rapid Bootstrapping algorithm of 1000 replicates under the GTRCAT model [38]. Samples with only ITS sequences were then added to the data matrix and the final phylogenetic tree was inferred using the same approach but with the constraint tree option using the initial multi-locus phylogeny.

PartitionFinder 2 [39] was used to determine which evolutionary models should be assigned to the partitions for the Bayesian analysis whereas the codon positions were partitioned individually. The following substitution models were applied: GTR + I + gamma for ITS, the second codon positions of *MCM7*, *RPB1*, *RPB2* and *TEF-1α* exons; GTR + gamma for the third codon positions of *MCM7* and *TEF-1α* exons; GTR + I for the first codon positions of *RPB2* and *TEF-1α* exons; HKY + I + gamma for the first codon positions of *MCM7* and *RPB1* exons; HKY + gamma for *RPB1* and *TEF-1α* introns; HKY + I for the third codon positions of *RPB1* and *RPB2* exons. Four separate Markov chain Monte Carlo (MCMC) runs were performed, and each chain was run for 50 million generations and sampled every 10.000th generation. 25% of the trees from each run were excluded as a burn-in and the four runs were merged using the sump function. A 50% majority rule consensus tree was generated using the sumt function with default parameters.

The phylogenetic trees were merged using TreeGraph 2 [40] and edited using Inkscape 0.92.

### Nomenclature

The electronic version of this article in Portable Document Format (PDF) in a work with an ISSN or ISBN will represent a published work according to the International Code of Nomenclature for algae, fungi, and plants, and hence the new names contained in the electronic publication of a PLOS article are effectively published under that Code from the electronic edition alone, so there is no longer any need to provide printed copies.

In addition, new names contained in this work have been submitted to MycoBank from where they will be made available to the Global Names Index. The unique MycoBank number can be resolved and the associated information viewed through any standard web browser by appending the MycoBank number contained in this publication to the prefix http://www.mycobank.org/MB/. The online version of this work is archived and available from the following digital repository: LOCKSS.

## Results

A total of 101 new consensus sequences from five nuclear markers of specimens of *Russula* spp. belonging to the subsect. *Roseinae* recently collected in Panama were generated in the context of this study (Table 1). 22 new ITS sequences, 20 new *RPB1* sequences, 21 new *RPB2* sequences, 21 new *MCM7* sequences and 17 new *TEF1-α* sequences were uploaded to the public database GenBank® (http://www.ncbi.nlm.nih.gov/). The final dataset was complemented by 322 sequences retrieved from public data bases (S2 Table).

**Table 1:** Sequences newly generated for this study.

Species	Voucher no.	ITS	*MCM7*	*RPB1*	*RPB2*	*TEF-1α*
*R*. *cynorhodon* (holotype)	FH-18-117	MW058812	MW110912	MW315763	MW197755	MW297195
*R*. *cynorhodon*	FH-18-118	MW058809	MW110909	MW315764	MW197752	---
*R*. *cynorhodon*	FH-18-052	MW058811	MW110911	MW315762	MW197754	MW297193
*R*. *cynorhodon*	FH-18-036	MW058810	MW110910	MW315765	MW197753	MW297190
*R*. *cynorhodon*	FH-18-032	MW058813	MW110913	MW315761	MW197756	MW297189
*R*. *zephyrovelutipes* (holotype)	FH-18-116	MW058806	MW110906	MW315769	MW197749	MW297194
*R*. *zephyrovelutipes*	FH-18-051	MW058807	MW110907	MW315771	MW197750	MW297192
*R*. *zephyrovelutipes*	FH-18-050	MW058805	MW110905	MW315770	MW197748	MW297191
*R*. *oreomunneae* (holotype)	FH-18-151	MW058804	MW110904	MW315766	MW197747	MW297196
*R*. *oreomunneae*	AC190	KM594825*	MW110923	MW315767	MW197766	MW297182
*R*. *oreomunneae*	AC614	MW084355	MW110924	MW315768	MW197767	---
*R*. *cornicolor* (holotype)	FH-18-154	MW058808	MW110908	MW315756	MW197751	MW297197
*R*. *cornicolor*	AC494	MW084356	MW110914	MW315752	MW197757	MW297183
*R*. *cornicolor*	AC561	MW084357	MW110915	MW315757	MW197758	---
*R*. *cornicolor*	AC449	MW084358	MW110916	MW315753	MW197759	MW297184
*R*. *cornicolor*	AC091	KM594796*	MW110917	---	MW197760	MW297185
*R*. *cornicolor*	AC629	MW084359	MW110918	MW315754	MW197761	MW297186
*R*. *cornicolor*	AC518	MW084360	MW110919	MW315758	MW197762	MW297187
*R*. *cornicolor*	AC056	KM594788*	MW110920	MW315759	MW197763	---
*R*. *cornicolor*	AC064	KM594795*	MW110921	MW315755	MW197764	MW297188
*R*. *cornicolor*	AC167	KM594813*	MW110922	MW315760	MW197765	MW297181
*R*. *aurora* f. *armeniaca* (holotype)	R257	MW079511	---	---	---	---
*R*. *aurora* var. *gemella* (holotype)	RU 03081002	MW079512	---	---	---	---
*R*. *roseoalbescens* (holotype)	RU 03101001	MW079513	---	---	---	---
*R*. *spurcata* (holotype)	RU 02101001	MW079514	---	---	---	---

* Asterisks mark exceptions for which sequence data was retrieved from GenBank.

**Fig 1:**
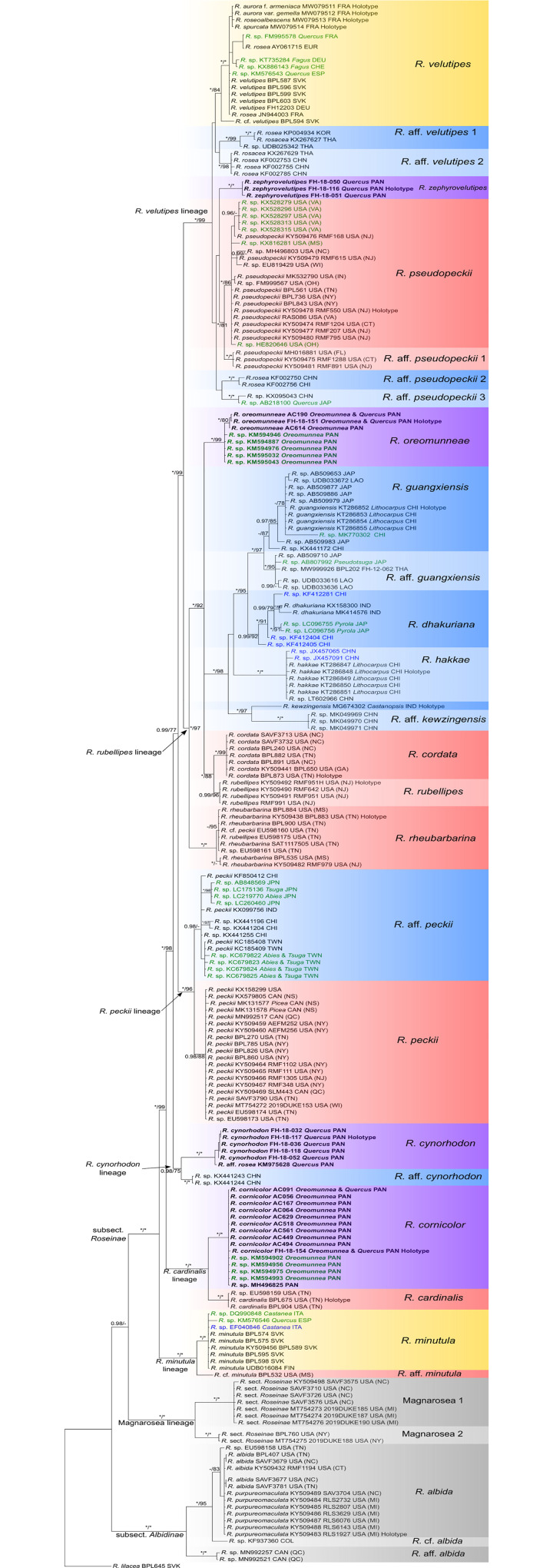
Fifty percent majority-rule Bayesian cladogram of the Roseinae clade from a concatenated data matrix of ITS, rpb1, rpb2, tef1, and mcm7. BI posterior probabilities are followed by ML values derived from 1000 bootstrapping replicates. Full support is indicated by an asterisk (*). The highlighting of the clades corresponds to the geographic origin of the collections: yellow: Europe, blue: Asia, purple: Panama, red: North America. Specimen labels in green indicate sequences derived from plant roots and blue indicates sequences derived from soil. Sequences of newly described species are in bold. GenBank accession numbers are indicated for collections of which only ITS sequences were available. Countries are abbreviated by ISO codes (https://www.iso.org/iso-3166-country-codes.html), US and Canadian states by postal codes (http://www.icq.eps.harvard.edu/ICQpost.html). Associated plants are labeled where known with high confidence.

The topologies of the phylogenetic trees resulting from Bayesian and Maximum likelihood analyses of the multi locus alignment were identical except for one branch, including *Russula rheubarbarina* Looney (Fig 1).

The phylogenetic analysis revealed that the 22 Panamanian collections in subsect. *Roseinae* form four well-supported species clades placed within four unrelated lineages (Fig 1). Four new species names are formally proposed for all four species clades and detailed morphological descriptions are provided in the Taxonomy section (below).

*Russula cornicolor* Manz & F. Hampe sp. nov. is a sister species of the North American *Russula cardinalis* Looney. These two species form the independent *Russula cardinalis*-lineage.

*Russula cynorhodon* Manz & F. Hampe sp. nov. forms a small supported lineage together with a potentially undescribed species represented by two samples from China.

The clade corresponding to *R*. *oreomunneae* Manz, F. Hampe & Corrales sp. nov. is placed in a polytomy within the *R*. *rubellipes* lineage. This polytomy has three distinct lineages, one with the single species *R*. *oreomunneae*, a second with at least six Asian species and a third with the North American species *R*. *cordata* Looney and *R*. *rubellipes* Fatto.

For *Russula zephyrovelutipes* Manz & F. Hampe sp. nov., no closely related known species were detected in our analyses.

Our study revealed a close relationship of Panamanian species to either North American or Southeast Asian species of subsect. *Roseinae*, but there is no evidence that any Panamanian species is closely related to any European species. Only *R*. *zephyrovelutipes*, together with the North American species *Russula pseudopeckii* Fatto and several undescribed Asian species, is included in a larger clade which also contains the European *R*. *velutipes*.

Since the ITS sequences retrieved from holotypes of the European taxa *R*. *aurora* f. *armeniaca*, *R*. *aurora* var. *gemella*, *R*. *roseoalbescens* and *R*. *spurcata* all clustered within the *Russula velutipes* clade, they are probably conspecific with this species.

### Taxonomy

**Fig 2:**
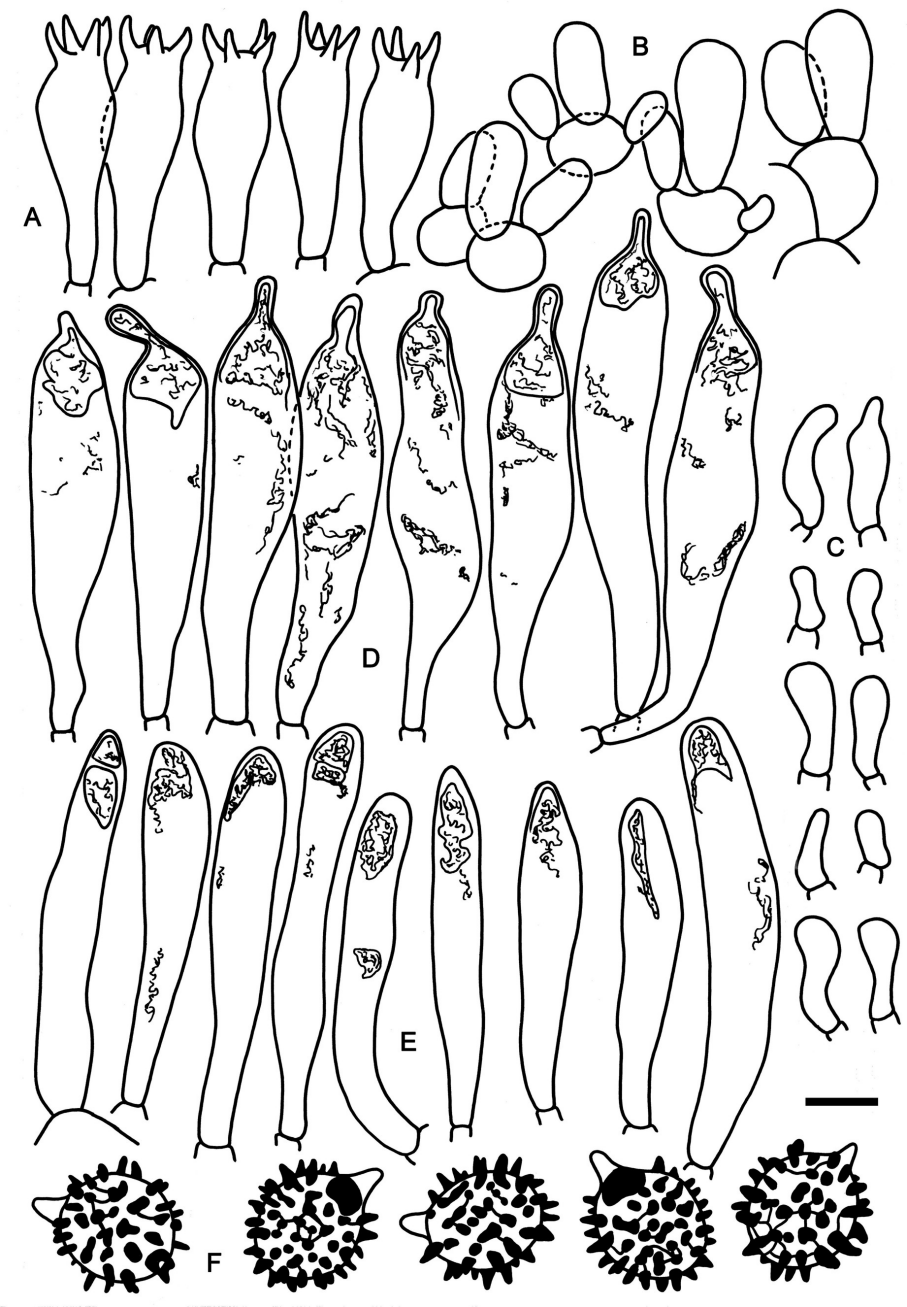
Hymenial elements of *Russula cornicolor* (holotype FH-18-154). A: Basidia. B: Basidiola. C: Marginal cells. D: Hymenial cystidia. E: Hymenial cystidia near the lamellae edges. F: Spores as seen in Melzer’s reagent. Scale bar = 10 μm, but only 5 μm for spores.

**Fig 3:**
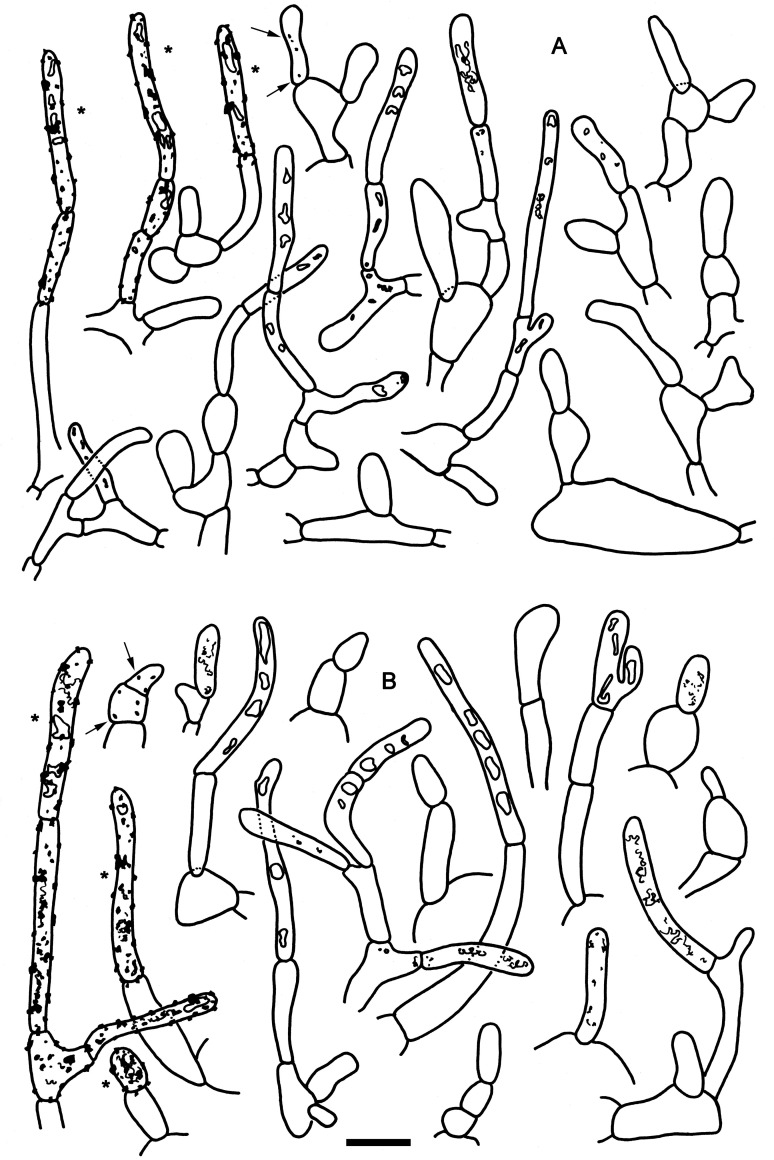
Elements of the pileipellis of *Russula cornicolor* (holotype FH-18-154). A: Hyphal terminations and primordial hyphae with contents near the pileus margin. B: Hyphal terminations and primordial hyphae with contents near the pileus center. Asterisks (*) mark primordial hyphae observed after carbolfuchsin treatment with acid resistant incrustations. Arrows indicate inclusions. Scale bar = 10 μm.

***Russula cornicolor*** Manz & F. Hampe, **sp. nov.** (Figs 2, 3, 10F, 11E and 11F)

MycoBank: MB837499

**Holotype:** Panama, Chiriquí, Reserva Forestal Fortuna, 08°45’11.1’’N; 82°14’22.56’’W, alt. 1207 m, tropical montane mixed forest with *Quercus* spp. and *Oreomunnea mexicana*, 29 Jun 2018, F. Hampe, C. Manz & T. A. Hofmann FH-18-154 (Holotype: M-0141377; Isotype: UCH11720).

**Etymology:** Referring to the colour of pileus and stipe that is similar to the colour of the fruits of *Cornus mas* L.

**Pileus** small to medium-sized, 10–57 mm diam., convex with incurved margin when young, with age becoming plane or depressed at the center, almost infundibuliform with decurved margin; margin at first even, up to 4 mm striate when old; cuticle dry, smooth to slightly rugulose, towards the margin finely areolate, minutely pruinose at the center, peeling to ½ of the radius, near the margin dull red (10C4), dusty pink (10C5), towards the center pale red (10A3), grey-red (10B5, 10D4), dull red (10C4), dusty pink (10C5), brown-red (10D6), raspberry (10D7), strawberry (10D8), sometimes with small whitish discoloured spots. **Lamellae** 2–5 mm wide, thin, moderately distant, 9 at 1 cm near the pileus margin, adnexed, adnate or subdecurrent, white to pale cream, with indistinct anastomoses near the bases; lamellulae absent; edges entire and concolorous. **Stipe** 13–45 × 3–12 mm, cylindrical, sometimes bent or slightly widened towards the base, pastel red (10A4) to bright red (10A5) from the base up to at least half-length of the stipe, white near the lamellae, smooth to slightly rugose, dry, medulla cottony, stuffed and with age becoming hollow. **Context** white, fragile, firm in young fruiting bodies, unchanging when damaged, 1–2 mm thick at half of pileus diameter. **Macrochemical reactions:** guaiac after 5 seconds negative on both stipe and lamellae, FeSO_4_ almost negative (slightly orange), sulfo vanillin eosin red. **Taste** mild. **Odour** inconspicuous or pleasant. **Spore print** not observed.

**Spores** (6.3–)7.2–7.6–8.0(–8.8) × (5.6–)6.1–6.5–6.9(–7.6) μm, broadly ellipsoid, Q = (1.05–)1.12–1.17–1.22(–1.35); ornamentation of large, prominent, moderately distant [(3–)4–6(–8) in a 3 μm circle] amyloid spines, (0.9–)1.1–1.5(–1.9) μm high, mainly isolated, rarely fused [0(–2) fusions in the circle], connected by often frequent, fine lines sometimes disconnecting into punctations that are hardly visible in light microscope [(0–)1–4(–7) lines in the circle], suprahilar spot moderately large, amyloid. **Basidia** (25.0–)28.1–31.6–35.1(–39.5) × (9.5–)10.6–11.4–12.2(–14.5) μm, broadly clavate or obpyriform, 4-spored; basidiola cylindrical or clavate, ca. 6.5–10 μm wide. **Hymenial cystidia** (48.5–)56.0–64.2–72.4(–94.5) × (7–)9.9–11.9–13.9(–18) μm, clavate, frequently slightly constricted near the apical part, rarely curved near the base, apically obtuse, occasionally acute, mainly with a 3–9(–14.5) μm long appendage, originating in subhymenium and somewhat protruding over basidia, thin-walled; contents in Congo Red mainly dispersed with larger refringent inclusion in the apical part, not reacting to sulfo vanillin; cystidia near the lamellae edges numerous, smaller and narrower (24–)41.3–49.2–57.1(–65.5) × (4–)6.6–8.1–9.6(–13.5) μm, cylindrical or narrowly clavate, rarely subfusiform, obtuse or rarely acute and without an appendage. Lamellae edges sterile; **marginal cells** similar to basidiola, mainly clavate, often flexuous, (7–)12.6–17.3–22(–29) × (3.0–)3.8–4.8–5.8(–9.5) μm. **Pileipellis** with fine orthochromatic incrustations and slightly metachromatic cell walls in Cresyl Blue, not well delimited from the underlying context, 60–90 μm deep, suprapellis 12–27 μm deep, strongly gelatinized, composed of ascending to erect hyphal terminations, very loose towards the surface, relatively dense towards the context, embedded in a thick (up to 20 μm) extra gelatinous matter, well delimited from 44–77 μm deep, not gelatinized, dense subpellis of inflated (up to 23 μm) cells forming a pseudoparenchymatic structure and near trama passing to a layer of fibrillose hyphae. **Hyphal terminations** near the pileus margin composed of 1(–2) unbranched cells, stout and short, thin-walled, terminal cells (5.5–)8.4–11.7–15.0(–21.5) × (2.5–)3.2–3.9–4.6(–7) μm, cylindrical, ellipsoid or broadly clavate, rarely attenuated or irregularly shaped, apically obtuse, very rarely with some inclusions; subterminal cells frequently branched, more inflated, ca. 3–8.5 μm wide and frequently isodiametrical. Hyphal terminations near the pileus center distinctly shorter, terminal cells (4.5–)7.2–9.6–12.0(–19.5) × (2–)3.4–4.3–5.2(–7) μm, similar in shape, more frequently with dispersed inclusions; subterminal cells of similar structure. **Primordial hyphae** with abundant acid resistant incrustations, near the pileus margin 1–4-celled (av. 1.7), long and slender, sometimes flexuous, thin-walled, terminal cells (10.5–)14.9–20.3–25.7(–34.5) × (2.5–)2.8–3.2–3.6(–4.5) μm, cylindrical, contents mainly refingent, forming small patches, sometimes more heteromorphous and almost banded, less frequently dispersed, present in 1–3 (av. 1.2) cells. Primordial hyphae near the pileus center 1–3-celled (av. 1.2), wider, terminal cells (7–)11.0–27.7–24.4(–37) × (2.5–)3.3–4.0–4.7(–8) μm, contents in 1–2 (av. 1.1) cells. **Cystidioid or oleiferous hyphae** in the subpellis and the context absent.

**Additional material studied:** Panama. Chiriquí, Reserva Forestal Fortuna, Gualaca District, Alto Frio plot, 8˚39’25"N; 82˚12’54"W, alt. 1176 m, under *Oreomunnea mexicana* and *Quercus* sp., 22 May 2012, A. Corrales & C. Velásquez, AC190 (ARIZ); *ibid*. Honda watershed—Nitrof 54 plot, 8°45’22.8’’N; 82°14’47.1’’W, alt. 1326 m, monodominant stand of *Oreomunnea mexicana*, 11 Jan 2014, A. Corrales, AC614 (FLAS).

**Notes:** *Russula cornicolor* is the sister species of *Russula cardinalis* known from the Eastern United States. Both species have negative guaiac reactions on both, stipe and lamellae surfaces, an average spore length that is larger than seven micrometers and a trichodermal suprapellis structure near the pileus center. *Russula cardinalis* differs from *R*. *cornicolor* by thick-walled pleurocystidia. The terminal cells of the hyphae near the pileus margin are longer and narrower in *R*. *cardinalis* (Q-value 4–5) than those of *R*. *cornicolor* (Q-value 2.5–3.5), and terminal cells of primordial hyphae near the pileus margin are on average wider than three micrometers in *R*. *cardinalis*, which is the maximum width in *R*. *cornicolor*.

**Fig 4:**
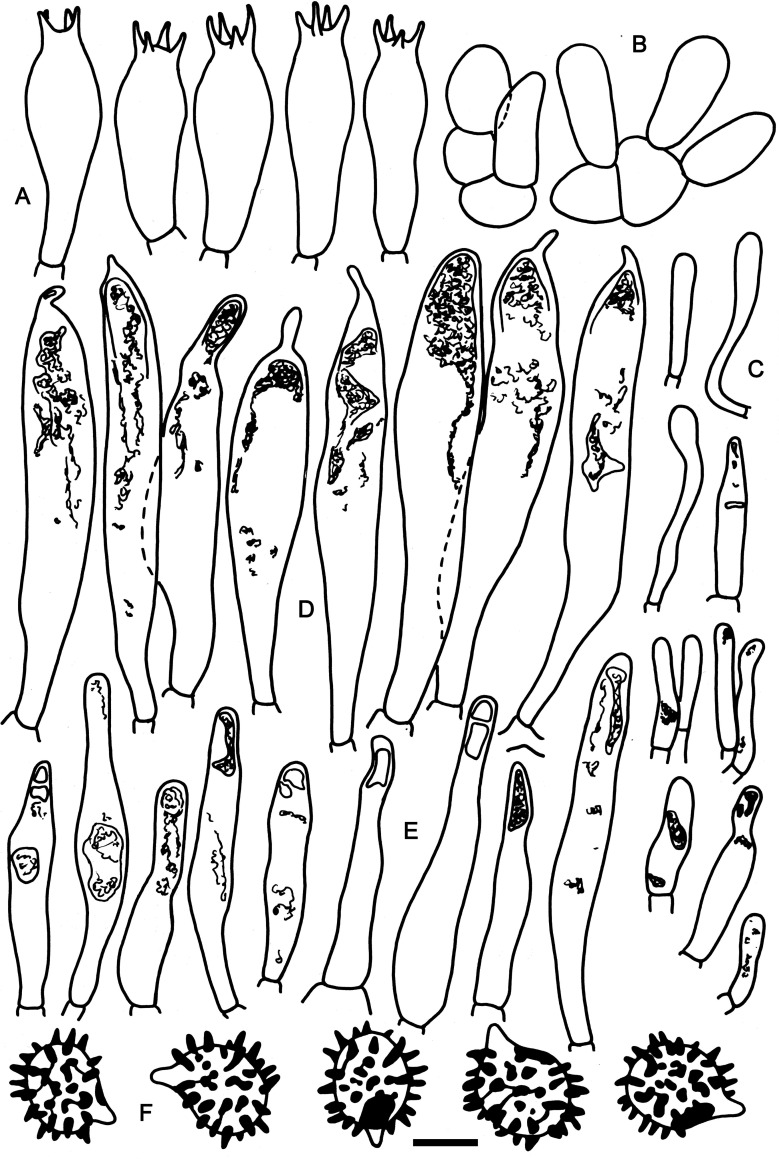
Hymenial elements of *Russula cynorhodon* (holotype FH-18-117). A: Basidia. B: Basidiola. C: Marginal cells. D: Hymenial cystidia. E: Hymenial cystidia near the lamellae edges. F: Spores as seen in Melzer’s reagent. Scale bar = 10 μm, but only 5 μm for spores.

**Fig 5:**
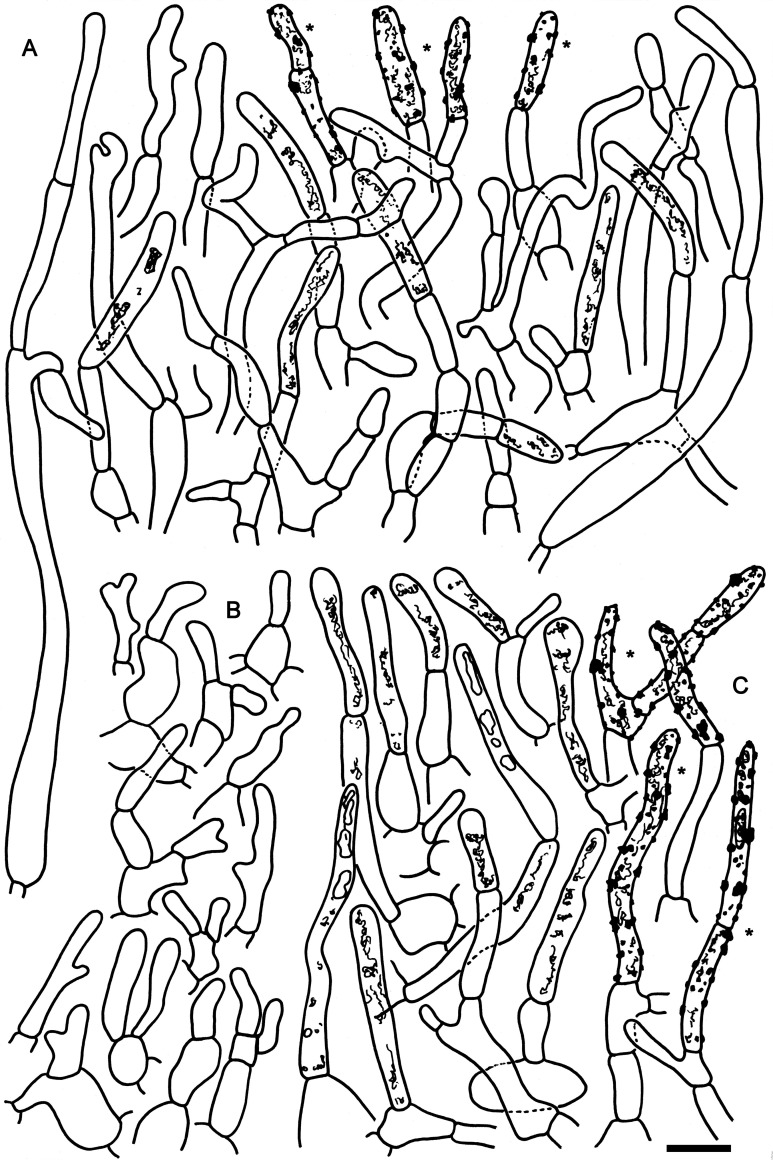
Elements of the pileipellis of *Russula cynorhodon* (holotype FH-18-117). A: Hyphal terminations and primordial hyphae (with contents) near the pileus margin. B: Hyphal terminations near the pileus center. C: Primordial hyphae near the pileus center. Asterisks (*) mark primordial hyphae observed after carbolfuchsin treatment with acid resistant incrustations. Scale bar = 10 μm.

***Russula cynorhodon*** Manz & F. Hampe, **sp. nov.** (Figs 4, 5, 10A–10C and 11A)

MycoBank: MB837496

**Holotype:** Panama, Chiriquí, Boquete, Jaramillo Arriba, El Musgo, 08°47’26.9’’N 82°24’34.4’’W, alt. 1669 m, mixed tropical montane forest with *Quercus* spp., 24 Jun 2018, F. Hampe, C. Manz & T. A. Hofmann FH-18-117 (Holotype: M-0141368; Isotype: UCH11713).

**Etymology:** Derived from the greek word for rose hips (fruits of *Rosa canina* L., gr. kynórodo) and referring to the colour of the pileus of young fruiting bodies.

**Pileus** small to medium-sized, 14–49 mm diam., hemisphaerical or convex when young, becoming depressed or almost infundibuliform at the center; margin even and up to 4 mm striate when old; cuticle dry, smooth, matt and velvety, peeling hardly to ¼ of the radius, near the margin pink white (9A2), pale red (9A3), pastel red (9A4), bright red (9A5), red (9A6), madder (9A7), dull red (9B4), rosy-cheeked (9B5), grey-red (9B6), coral red (9B7), towards the center pink white (9A2), pale red (9A3), pastel red (9A4), bright red (9A5), red (9A6), madder (9A7), vermilion (9A8), rosy-cheeked (9B5), grey-red (9B6), coral red (9B7), frequently with small pale yellow (4A3) or bright yellow (4A4) spots. Lamellae 2–4 mm wide, thin, moderately dense, 10–11 at 1 cm near the pileus margin, adnexed, white to yellow-white (4A2) or pale yellow (4A3), occasionally forked, especially near the stipe; lamellulae absent, edges entire and concolorous. **Stipe** 23–70 x 4–17 mm, mostly clavate and narrowing towards the apex, rarely attenuated towards the base, white and sometimes with a light pastel red flush near the base, smooth, medulla cottony, stuffed. **Context** white, fragile, unchanging when damaged. **Macrochemical reactions:** guaiac after 5 seconds negative on stipe but positive on lamellae (++), FeSO_4_ weak salmon orange, sulfo vanillin eosin red. **Taste** first mild, then slightly spicy. **Odour** inconspicuous. **Spore print** white (Ib) to pale cream (IIa).

**Spores** (6–)6.7–7.1–7.5(–8.1) x (5.1–)5.8–6.1–6.4(–7) μm, subglobose to broadly ellipsoid, Q = (1.04–)1.12–1.17–1.22(–1.35); ornamentation of isolated or clustered spines connected by low and short lines, amyloid spines moderately distant [(3–)4–6(–7) in a 3 μm diam. circle], normal to high (up to (0.8–)0.9–1.1–1.3(–1.8) μm high), occasionally fused in pairs or short ridges [(0–)1–2(–3) fusions in the circle] and frequently connected by short line connections [(0–)1–3(–5) lines in the circle], suprahilar spot moderately large, amyloid and sometimes slightly descending onto hilum. **Basidia** (23.5–)32–35.6–39(–47) x (9.5–)10.5–11.3–12(–14) μm, stout, clavate, 4-spored; basidiola cylindrical or clavate, ca. 7–10 μm wide. **Hymenial cystidia** (45–)57.5–68.7–79.5(–111) x (8–)10–11.4–13(–16) μm, clavate or rarely fusiform, apically obtuse or rarely acute, frequently with a (2–)3–9(–16) μm long appendage (mostly absent in one collection), originating in subhymenium and protruding ca. 30 μm over basidia, thin-walled; contents mainly dispersed with larger refringent inclusion in the apical part, not reacting to sulfo vanillin; cystidia near the lamellae edges numerous, smaller and narrower, (31–)37.5–46.7–55.5(–70) x (4.5–)6–7.7–9.5(–12.5) μm, irregularly shaped with constrictions or partly inflated, rarely with an appendage, occasionally with 1–2(–3) secondary septa. Lamellae edges sterile; **marginal cells** similar to hymenial cystidia, but shorter and narrower, very variable in shape: cylindrical, clavate, fusiform, flexuous or with constrictions, without or frequently with contents similar to hymenial cystidia, (11–)14.5–18.6–23(–32) x (2.5–)3–4.1–5(–6.5) μm. **Pileipellis** with very weak metachromatic reaction in Cresyl Blue, not well delimited from the underlying context, 50–70 μm deep, suprapellis 15–23 μm deep, composed of ascending hyphal terminations, very loose towards the surface, relatively dense towards the interior, embedded in a thick (up to 20 μm) extra gelatinous matter, well delimited from 30–50 μm deep, not gelatinized, dense subpellis of inflated (up to 12 μm) cells forming a pseudoparenchymatic structure and near trama passing to a layer of fibrillose hyphae. **Hyphal terminations** near the pileus margin composed of 1–3(–4) unbranched cells, sometimes twisted or bent, thin-walled, terminal cells (6–)9.5–15.2–21(–4 AC4493.5) x (2.5–)3–3.8–4.5(–6) μm, mostly cylindrical, sometimes attenuated or clavate, apically obtuse; subterminal cells usually unbranched, equally shaped and of similar size. Hyphal terminations near the pileus center of 1(–2) unbranched cells and distinctly shorter; terminal cells (4–)8.5–11.5–14.5(–25) x (2–)3–3.7–4.5(–6) μm, equally shaped, subterminal cells frequently branched and more irregularly shaped. **Primordial hyphae** with abundant acid resistant incrustations, near the pileus margin 1–5-celled (av. 2.3), straight or slightly flexuous, thin-walled; terminal cells (7–)13–19.4–26(–46.5) x (2.5–)3.5–3.8–4.5(–5) μm, cylindrical, apically obtuse with dispersed oily contents in 1–4 (av. 1.5) cells. Primordial hyphae near the pileus center 1–4-celled (av. 1.5), terminal cells (7–)10–18–26(–45.5) x (2.5–)3.5–4.3–5(–6) μm, contents in 1–3 (av. 1.3) cells. **Cystidioid hyphae** not observed. **Oleiferous hyphae** very dispersed in the context.

**Additional material studied:** Panama. Chiriquí, Las Nubes, Alto Chiquero, close to the house of ANAM, alt. 1770 m, on soil close to *Quercus* sp., 26 Aug 2007, M. Piepenbring and undergraduate students MP3945 (M-0141101); Chiriquí, Boquete, Jaramillo Arriba, El Musgo, 08°47’26.9’’N 82°24’34.4’’W, alt. 1669 m, tropical montane mixed forest with *Quercus*, 15 Jun 2018, F. Hampe, C. Manz & T. A. Hofmann FH-18-032 (M-0141372, duplicate: UCH11716); *ibid*. FH-18-036 (M-0141371, duplicate: UCH11715); *ibid*. FH-18-052 (M-0141370, duplicate: UCH11740); *ibid*. 24 Jun 2018, F. Hampe, C. Manz & T. A. Hofmann FH-18-118 (M-0141369, duplicate: UCH 11714).

**Notes:** *Russula cynorhodon* is characterised by a trichodermal suprapellis structure in both the margin and center and a positive guaiac reaction on the gills. There are several species that are microscopically very similar, but they belong to different phylogenetic lineages within *Russula* subsect. *Roseinae*. For example, *R*. *pseudopeckii*, *R*. *rheubarbarina and R*. *zephyrovelutipes* are similar to *R*. *cynorhodon*, but the latter differs by an average spore size that is longer than seven micrometers and by a spore ornamentation that is higher than one micrometer.

In our phylogenetic analysis, we included the Panamanian collection MP3945 that was previously identified as *Russula mexicana* Burl. [11]. This collection was considered the only record of this species in Panama. *Russula mexicana* is a red-capped species with an acrid taste originally described from Mexico [41] and misidentified in the case of the Panamanian collection. Based on our phylogenetic and morphological study, *R*. *mexicana* has to be removed from the short list of Panamanian *Russula* spp.

**Fig 6:**
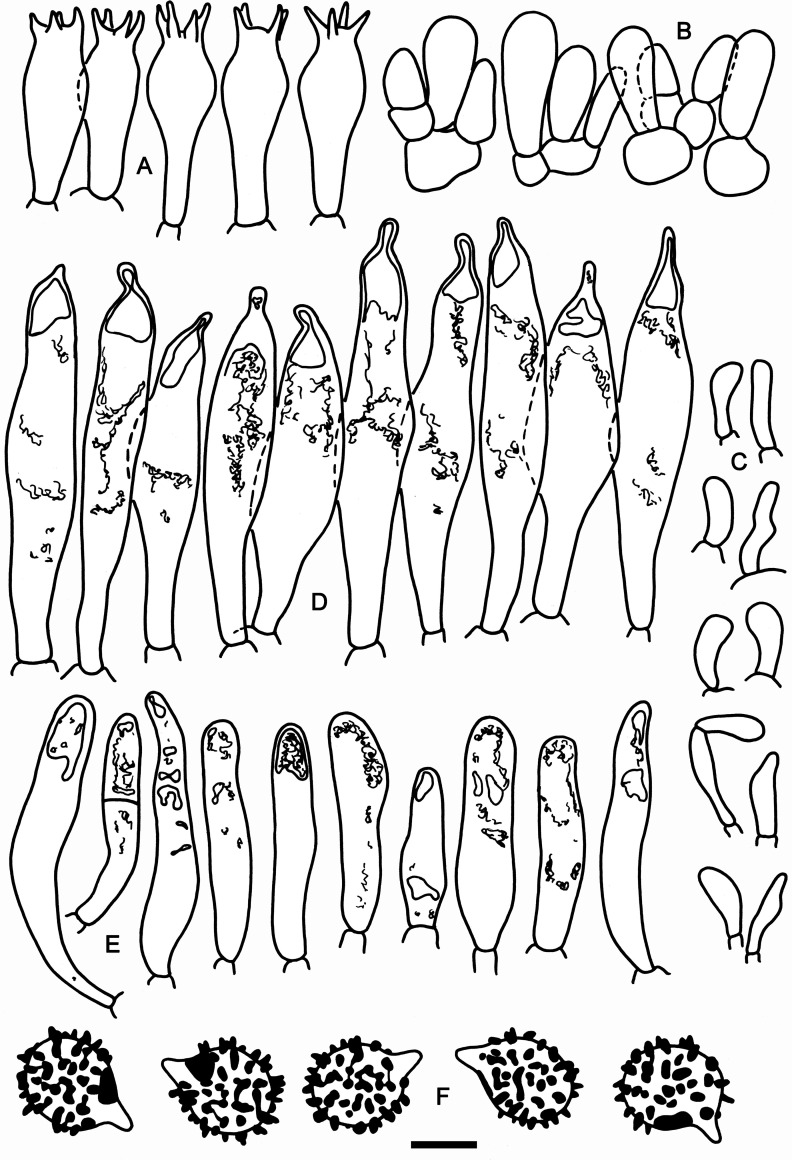
Hymenial elements of *Russula oreomunneae* (holotype FH-18-151). A: Basidia. B: Basidiola. C: Marginal cells. D: Hymenial cystidia. E: Hymenial cystidia near the lamellae edges. F: Spores as seen in Melzer’s reagent. Scale bar = 10 μm, but only 5 μm for spores.

**Fig 7:**
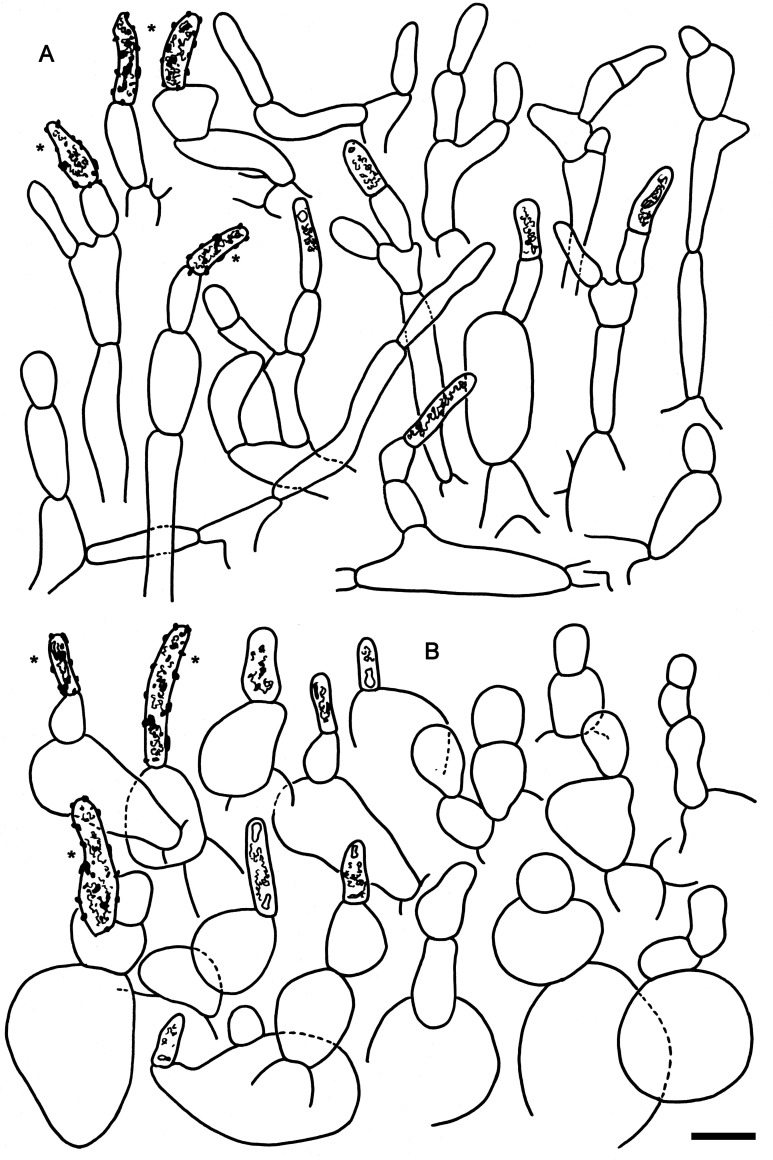
Elements of the pileipellis of *Russula oreomunneae* (holotype FH-18-151). A: Hyphal terminations and primordial hyphae with contents near the pileus margin. B: Hyphal terminations and primordial hyphae with contents near the pileus center. Asterisks (*) mark primordial hyphae observed after carbolfuchsin treatment with acid resistant incrustations. Scale bar = 10 μm.

***Russula oreomunneae*** Manz, F. Hampe & Corrales, **sp. nov.** (Figs 6, 7, 10E, 11C and 11D)

MycoBank: MB837498

**Holotype:** Panama, Chiriquí, Reserva Forestal Fortuna, 08°45’11.1’’N; 82°14’22.56’’W, alt. 1207 m, tropical montane mixed forest with *Quercus* and *Oreomunnea mexicana*, 29 Jun 2018, F. Hampe, C. Manz & T. A. Hofmann FH-18-151 (Holotype: M-0141376, Isotype: UCH11741).

**Etymology:** Referring to the ectomycorrhizal association with *Oreomunnea mexicana*.

**Pileus** small to medium-sized, 25–56 mm diam., plano-convex and with broad shallow central depression; margin distinctly crenate and up to 4 mm striate when old, decurved; cuticle descending up to 1 mm on gill edges, dry, smooth, matt, finely velutinous, towards the margin finely areolate, hardly peeling to ¼ of the radius, near the margin pale red (10A3), pastel red (10A4), bright red (10A5), red (10A6), morning red (10B4), grey-red (10B5, 10B6), towards the center bright red (10A5), brown-red (10C6, 10C7). Lamellae 2–4 mm wide, thin, moderately distant, 10–11 at 1 cm near the pileus margin, adnexed to adnate, white to pale cream (2A2), furcations and lamellulae absent, edges entire and mostly concolorous, near the pileus margin red from descending cuticle. **Stipe** 28–60 × 4–10 mm, cylindrical, mostly pastel red (10A4) or dull red (9B4) on white background, less frequently white with only a reddish tint, dry, smooth to slightly rugose, medulla cottony, stuffed. **Context** white, fragile, unchanging when damaged. **Macrochemical reactions:** guaiac after 5 seconds negative on stipe but positive on lamellae (++), FeSO_4_ orange, sulfo vanillin eosin red. **Taste** mild. **Odour** inconspicuous. **Spore print** not observed.

**Spores** (6–)6.4–6.8–7.2(–7.9) × (4.9–)5.4–5.8–6.2(–6.6) μm, subglobose to broadly ellipsoid, Q = (1.05–)1.13–1.18–1.23(–1.31); ornamentation of moderately large, dense to very dense [(7–)8–10(–11) in a 3 μm diam. circle], amyloid spines, (0.7–)0.8–0.9–1.0(–1.2) μm high, clustered in pairs or short chains, often forming crests or wings [(1–)2–4(–5) fusions in the circle], with rare short line connections [(0–)1–2(–3) lines in the circle], isolated spines occasional to rare, suprahilar spot moderately large, amyloid. **Basidia** (19.5–)25.7–29.7–33.7(–41) × (8.5–)9.4–10.2–11.0(–12) μm, stout, broadly clavate or obpyriform, 4-spored; basidiola cylindrical or clavate, ca. 6–8 μm wide. **Hymenial cystidia** (50–)58.5–67–75.5(–93.5) × (7.0–)8.8–10.8–12.8(–15.0) μm, cylindrical or fusiform, rarely clavate, apically obtuse or acute, with a (1.5–)4–13(–17.5) μm long appendage, originating in subhymenium and somewhat protruding over basidia, thin-walled, numerous; contents mainly dispersed, with large, refringent inclusion in the apical part, not reacting to sulfo vanillin; cystidia near the lamellae edges numerous, smaller and narrower, (23–)31.4–40.4–49.4(–64.5) × (5–)5.7–7.2–8.7(–12.5) μm mostly cylindrical, less frequently clavate or attenuated, rarely inflated, apically obtuse and without an appendage, rarely with secondary septa. Lamellae edges sterile; **marginal cells** mainly clavate, often flexuous, (6.5–)10.2–13.9–17.6(–23) × (2.5–)–3.1–4.2–5.3(–7) μm. **Pileipellis** orthochromatic and finely incrusted in Cresyl Blue, well delimited from the underlying context, 55–84 μm deep, suprapellis 15–24 μm deep, strongly gelatinized near the margin trichoderm and composed of ascending hyphal terminations, near the center hymeniderm and composed of erect hyphal terminations, embedded in a thin (up to 6 μm) extra gelatinous matter, well delimited from 40–60 μm deep, not gelatinized, dense subpellis of inflated (up to 22 μm) cells forming a pseudoparenchymatic structure and sharply separated from the trama by a layer of horizontally arranged hyphae. **Hyphal terminations** near the pileus margin composed of 1–2(–4) unbranched cells, sometimes flexuous, thin-walled, terminal cells (4–)9.7–13.6–17.5(–26.5) × (3–)3.6–4.3–5.0(–7) μm, cylindrical or clavate, rarely attenuated, apically obtuse; subterminal cells usually unbranched, equal in size or sometimes more inflated or longer. Hyphal terminations near the pileus center of distinctly shorter and wider cells; terminal cells (4.5–)6.7–9.1–11.5(–16) × (3.5–)4.4–5.7–7.0(–9.0) μm, ovoid, ellipsoid, pyriform or globose, subterminal cells unbranched and frequently more inflated. **Primordial hyphae** with abundant acid resistant incrustations, near the pileus margin 1–4-celled (av. 2.2) before branching, straight or slightly flexuous, thin-walled; terminal cells (8.0–)10.8–15.6–20.4(–31.0) × (2.5–)3.3–3.9–4.5(–5.0) μm, cylindrical, apically obtuse, rarely narrowed, with dispersed oily contents in the terminal cells only. Primordial hyphae near the pileus center often shorter, 1–3-celled (av. 1.3), terminal cells (8–)–10.2–15.3–20.4(–26.5) × (2.5–)3.1–5–6.9(–12) μm, dispersed oily contents in terminal cell only. **Cystidioid hyphae** not observed. **Oleiferous hyphae** very dispersed in the context.

**Additional material studied:** Panama. Chiriquí, Reserva Forestal Fortuna, Honda watershed, Trail to Honda B plot, 8˚45’20"N; 82˚13’07"W, alt. 1200 m, monodominant stand of *Oreomunnea mexicana*, 19 Mar 2012, A. Corrales & C. Velásquez, AC056 (ARIZ); *ibid*. Honda B plot, 8˚45’20"N; 82˚13’07"W, alt. 1266 m, monodominant stand of *O*. *mexicana*, 09 Apr 2012, A. Corrales & C. Velásquez, AC064 (ARIZ); *ibid*. Honda A plot, 8˚45’12"N; 82˚13’08"W, alt. 1175 m, under *O*. *mexicana* and *Quercus* sp., 13 Apr 2012, A. Corrales, M. Rodríguez & C. Velásquez, AC091 (ARIZ); *ibid*. Honda B plot, 8˚45’20"N; 82˚13’07"W, alt. 1266 m, monodominant stand of *O*. *mexicana*, 16 May 2012, A. Corrales & C. Velásquez, AC167 (ARIZ); *ibid*. Nitrof 56 plot, out of transect, 8°45’15.6’’N; 82°14’51.4’’W, alt. 1291 m, monodominant stand of *O*. *mexicana*, 12 Oct 2013, A. Corrales, AC449 (FLAS); *ibid*. Nitrof 58 plot, out of transect, 8°45’9.1’’N; 82°14’50.40’’W, alt. 1239 m, monodominant stand of *O*. *mexicana*, 30 Oct 2013, A. Corrales, AC494 (FLAS); *ibid*. Nitrof 55 plot, 8°45’19.6’’N; 82°14’56.8’’W, alt. 1312 m, monodominant stand of *O*. *mexicana*, 20 Nov 2013, A. Corrales, AC518 (FLAS); *ibid*. Nitrof 54 plot, 8°45’22.8’’N; 82°14’47.1’’W, alt. 1326 m, monodominant stand of *O*. *mexicana*, 10 Dec 2013, A. Corrales, AC561 (FLAS); *ibid*. Nitrof 58 plot, 8°45’9.1’’N; 82°14’50.40’’W, alt. 1239 m, monodominant stand of *O*. *mexicana*, 13 Jan 2014, A. Corrales, AC629 (FLAS).

**Notes:** *Russula oreomunneae* is morphologically and phylogenetically closely related to *Russula cordata* and *Russula rubellipes*. All of these species have a suprapellis with inflated elements forming an epithelium near the pileus center. *Russula oreomunneae* typically presents a “snow-man” type of hyphal terminations in the pileus center, i.e., hyphal terminations are composed of several globose cells that are strongly constricted at the septa and gradually smaller towards the apex. *Russula oreomunneae* differs from *R*. *cordata* by the absence of lobate terminal cells near the pileus center. *Russula rubellipes* is similar to *R*. *oreomunneae* but differs by larger and more distant warts of the spore ornamentation, longer (up to 54 μm) terminal cells near the pileus margin compared to only up to 20.5 μm in *R*. *oreomunneae* and absent or very rare pleurocystidia, that are numerous in *R*. *oreomunneae* (Fig 11C).

**Fig 8:**
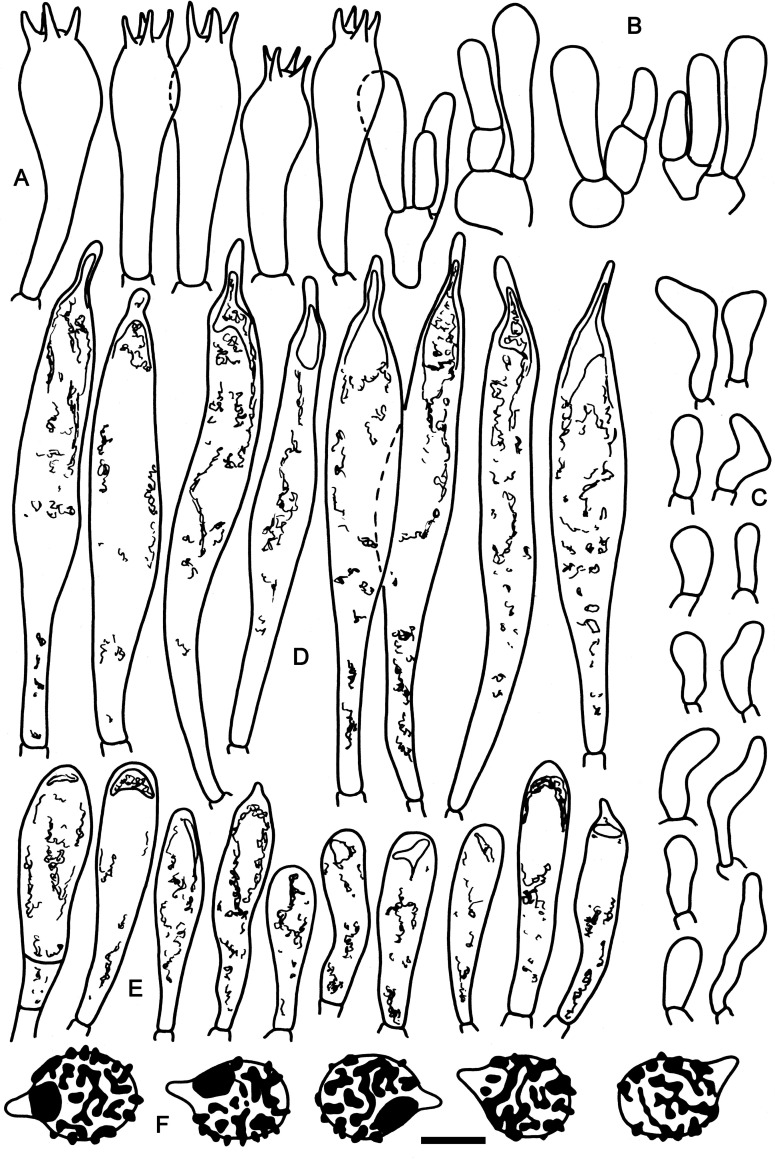
Hymenial elements of *Russula zephyrovelutipes* (holotype FH-18-116). A: Basidia. B: Basidiola. C: Marginal cells. D: Hymenial cystidia. E: Hymenial cystidia near the lamellae edges. F: Spores as seen in Melzer’s reagent. Scale bar = 10 μm, but only 5 μm for spores.

**Fig 9:**
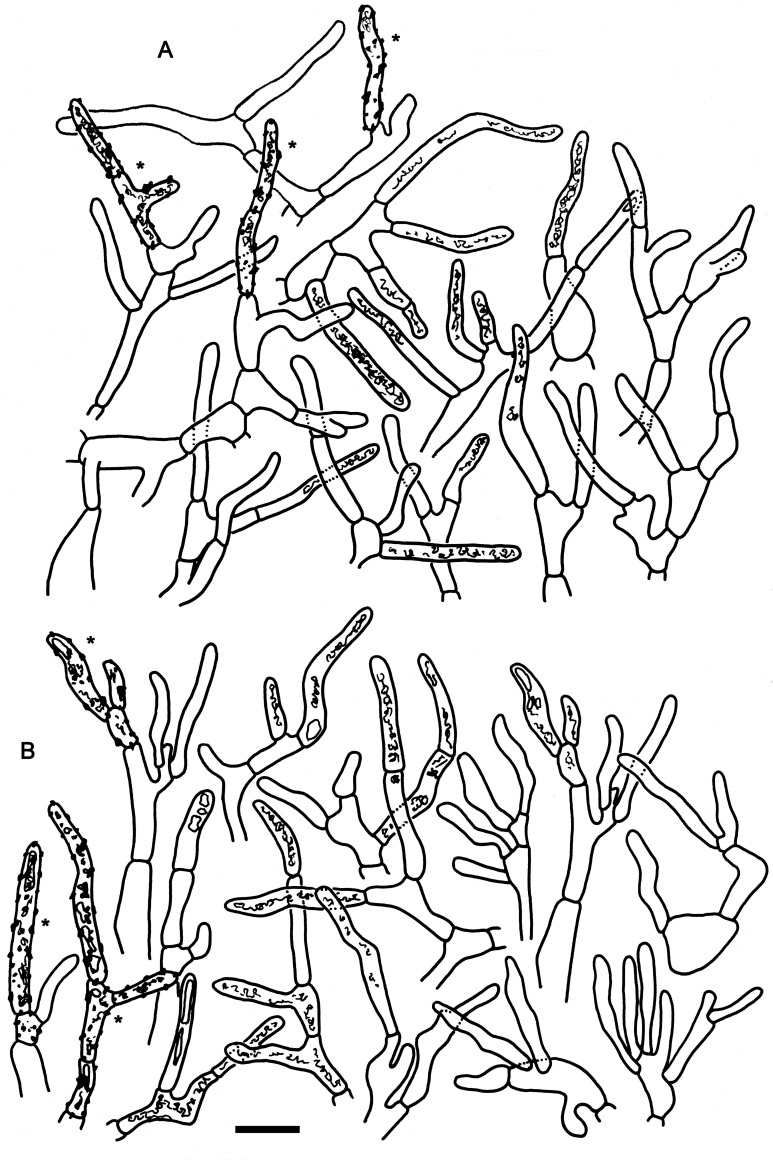
Elements of the pileipellis of *Russula zephyrovelutipes* (holotype FH-18-116). A: Hyphal terminations and primordial hyphae with contents near the pileus margin. B: Hyphal terminations and primordial hyphae with contents near the pileus center. Asterisks (*) mark primordial hyphae observed after carbolfuchsin treatment with acid resistant incrustations. Scale bar = 10 μm.

**Fig 10:**
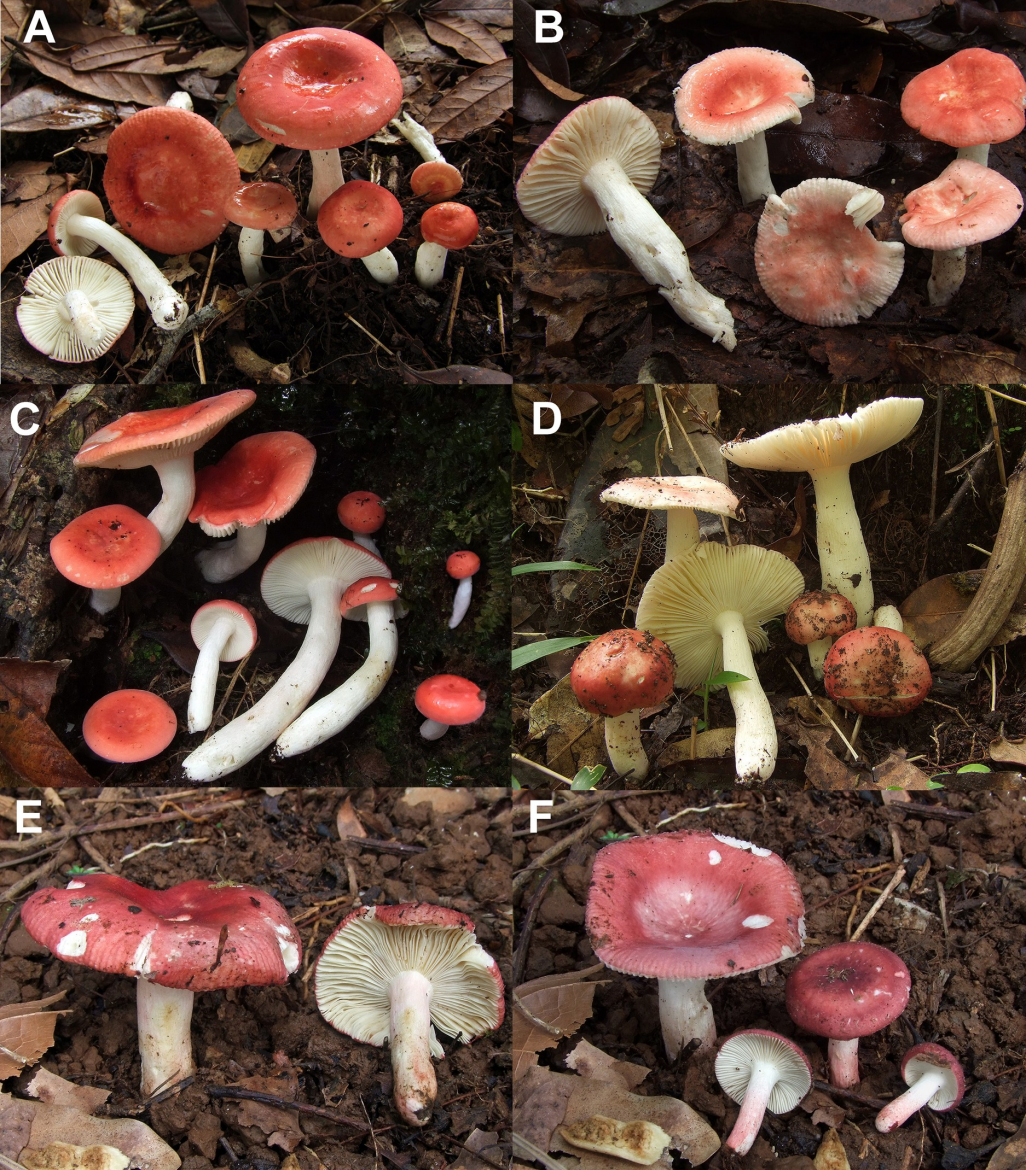
Field photographs of fruiting bodies of species described in this study. A: *Russula cynorhodon* (holotype FH-18-117). B: *Russula cynorhodon* (paratype FH-18-036). C: *Russula cynorhodon* (paratype FH-18-118). D: *Russula zephyrovelutipes* (holotype FH-18-116). E: *Russula oreomunneae* (holotype FH-18-151). F: *Russula cornicolor* (FH-18-154). All photographs: Felix Hampe.

**Fig 11:**
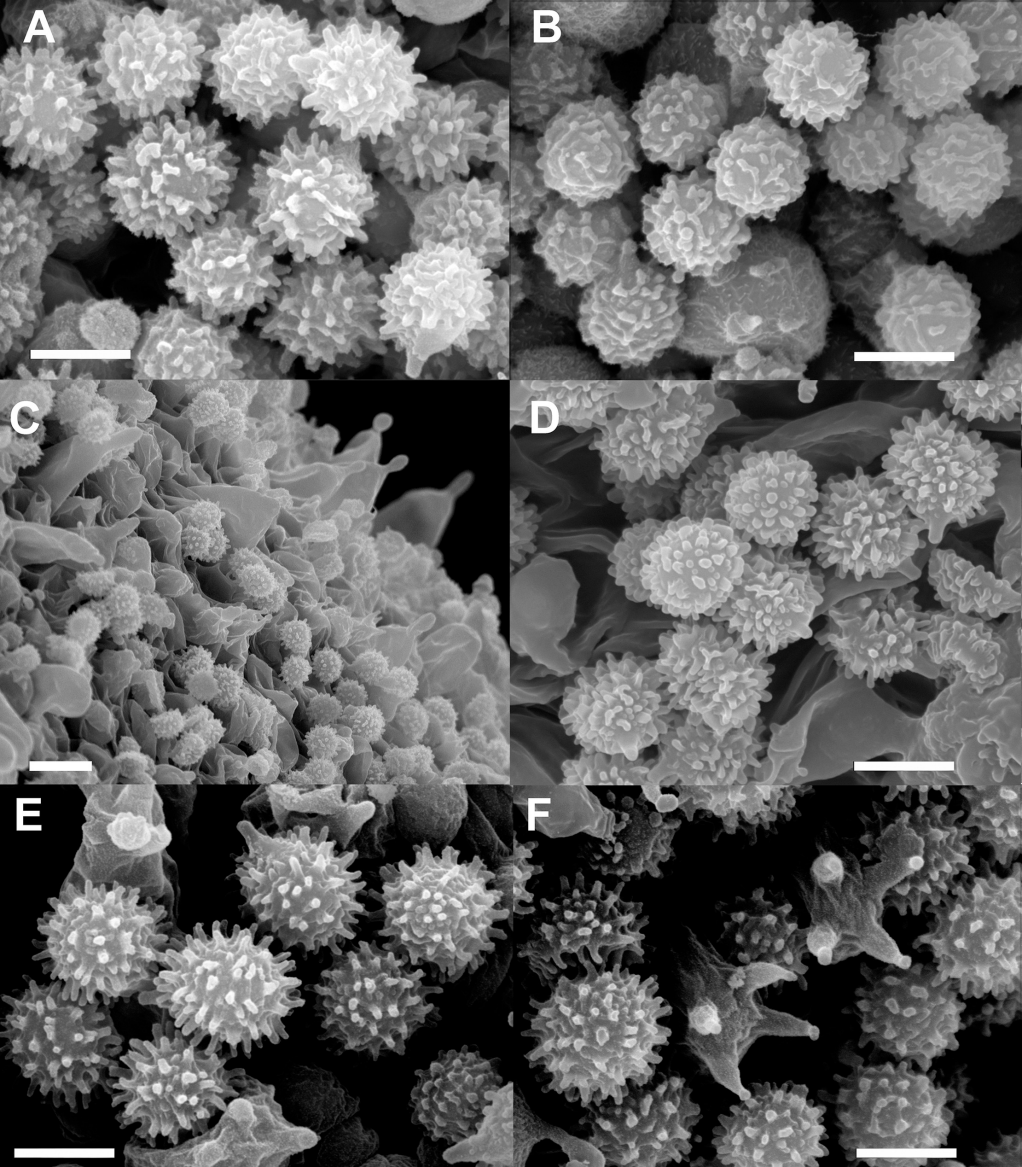
Scanning electron microscopic photographs of hymenial structures of species newly described in this study. A: Spores of *Russula cynorhodon* (holotype FH-18-117). B. Spores of *Russula zephyrovelutipes* (holotype FH-18-116). C: Pleurocystidia and spores of *Russula oreomunneae* (holotype FH-18-151). D: Spores of *Russula oreomunneae* (holotype FH-18-151). E: Spores of *Russula cornicolor* (holotype FH-18-154). F: Basidia and spores of *Russula cornicolor* (holotype FH-18-154). Scale bars = 10 μm.

***Russula zephyrovelutipes*** Manz & F. Hampe, **sp. nov.** (Figs 8, 9, 10D and 11B)

MycoBank: MB837497

**Holotype:** Panama, Chiriquí, Boquete, Jaramillo Arriba, El Musgo, 08°47’26.9’’N 82°24’34.4’’W, alt. 1669 m, tropical montane mixed forest with *Quercus*, 24 Jun 2018, F. Hampe, C. Manz & T. A. Hofmann FH-18-116 (Holotype: M-0141373; Isotype: UCH11717).

**Etymology:** Referring to the macromorphological similarity to *R*. *velutipes*, but with a western distribution. Zéfyros: west wind (used in Greek mythology).

**Pileus** small to medium-sized, 16–60 mm diam., hemispherical or convex when young, becoming plane and depressed at the center; margin at first even, becoming striate up to 5 mm when old; cuticle dry, smooth and matt, peeling to ¼–½ of the radius, near the margin orange white (5A2), pink white (9A2, 10A2), pale red (9A3, 10A3), pastel red (9A4, 10A4), bright red (9A5, 10A5), red (10A6), grey-red (9B6, 10B5, 10B6), brown-red (9C7, 10C6), towards the center pale red (9A3), pastel red (9A4), bright red (9A5), rosy-cheeked (9B5), grey-red (9B6), coral red (9B7), brown-red (9C7), dusty pink (10C5), raspberry (10D7) **Lamellae** 2–5 mm wide, thin, moderately dense, 9–12 at 1 cm near the pileus margin, adnexed, white to yellow-white (4A2), occasionally forked, especially near the stipe; lamellulae absent, edges entire and concolorous. **Stipe** 22–60 × 4–15 mm, mostly clavate and narrowing towards the apex, rarely cylindrical, white, sometimes with a light pastel red flush, smooth, medulla cottony, stuffed. **Context** white, fragile, unchanging when damaged. **Macrochemical reactions:** guaiac after 5 seconds negative on stipe but positive on lamellae (++), FeSO_4_ variable, orange to salmon orange, but negative in one collection, sulfo vanillin eosin red. **Taste** mild, slightly bitter after a while. **Odour** inconspicuous. **Spore print** not observed.

**Spores** (5.7–)6.3–6.7–7.1(–7.5) × (4.7–)5.3–5.7–6.1(–6.8) μm, subglobose to broadly ellipsoid, Q = (1.05–)1.08–1.18–1.28(–1.32); ornamentation of moderately large, moderately dense to dense [(2–)5–8(–11) in a 3 μm diam. circle], amyloid warts (0.4–)0.5–0.6–0.7(–0.8) μm high, clustered warts forming branched linear elements, sometimes even subreticulate, frequently fused into short or long ridges [(0–)1–4(–8) fusions in the circle], occasionally connected by short line connections [(0–)1–2(–4) lines in the circle]; suprahilar spot moderately large, amyloid. **Basidia** (28–)32.5–35.6–39(–42) × (10–)10.5–11.5–12.5(–14.5) μm, clavate or obpyriform, 4-spored; basidiola cylindrical or narrowly clavate, ca. 4.5–7 μm wide. **Hymenial cystidia** (54–)65–73.4–81.5(–88.5) × (8–)10–11.3–13(–16) μm, fusiform or subcylindrical, apically mostly acute, mainly with a (1–)2–9(–14) μm long appendage, originating in subhymenium and somewhat protruding over basidia, thin-walled; contents dominantly dispersed with refringent inclusion in apical part, not reacting to sulfo vanillin; near the lamellae edges numerous, smaller and narrower, (25.5–)37.5–48.3–59(–69) × (5–)7.5–9–10.5(–13) μm, cylindrical to clavate, obtuse, rarely with an appendage or secondary septa. Lamellae edges sterile; **marginal cells** mainly clavate, often flexuouse, (8–)12–17.5–23(–28) × (2–)3.5–4.5–5.5(–7) μm. **Pileipellis** orthochromatic in Cresyl Blue, not well delimited from the underlying context, 35–70 μm deep, suprapellis 15–25 μm deep, strongly gelatinised, composed of ascending to erect, loose hyphal terminations, embedded in a thick (up to 60 μm) extra gelatinous matter, well delimited from 20–45 μm deep, not gelatinized, dense subpellis of inflated (up to 11 μm) cells forming a pseudoparenchymatic structure and near the context passing to a layer of fibrillose hyphae. **Hyphal terminations** near the pileus margin usually composed of 1(–2) unbranched cells, occasionally slightly flexuous, thin-walled, terminal cells (10–)13.5–17.5–21.5(–28) × (2–)2–2.6–3(–4.5) μm, mostly cylindrical, sometimes attenuated, apically obtuse, rarely forked; subterminal cells mostly branched, more irregularly shaped, of similar size or wider. Hyphal terminations near the pileus center consist of a single unbranched cell; terminal cells slightly shorter, (5–)10.5–15–19.5(–26) × (1.5–)2–2.6–3(–4) μm, equally shaped, subterminal cells frequently branched several times, sometimes forming a broom-like appearance. **Primordial hyphae** with abundant acid resistant incrustations, near the pileus margin 1–2-celled (av. 1.3), sometimes slightly sinuous, thin-walled; terminal cells (5–)15.5–20.8–26.5(–33.5) × (2–)2.5–2.8–3.5(–4) μm, cylindrical or sometimes narrowed with obtuse tips, dispersed, oily contents usually only visible in the terminal cells (in one collection also in subterminal cells). Primordial hyphae near the pileus center 1–3-celled (av. 1.3), terminal cells (6–)13.5–18.6–24(–33) × (2–)2.5–2.9–3.5(–4) μm, contents more frequently present in subterminal cells. **Cystidioid hyphae** not observed. **Oleiferous hyphae** very dispersed in the context.

**Additional material studied:** Panama. Chiriquí, Boquete, Jaramillo Arriba, El Musgo, 08°47’26.9’’N 82°24’34.4’’W, alt. 1669 m, tropical montane mixed forest with *Quercus*, 15 Jun 2018, F. Hampe, C. Manz & T. A. Hofmann FH-18-050 (M-0141374, duplicate: UCH11719); *ibid*. FH-18-051 (M-0141375, duplicate: UCH11718).

**Notes:** *Russula zephyrovelutipes* is closely related to the morphologically similar species *Russula pseudopeckii*. Both species have a guaiac reaction that is negative on the stipe, but positive on the surfaces of lamellae, spores that are smaller than seven micrometers, a trichodermal suprapellis structure, and hyphae in the pileipellis with mostly branched subterminal cells. These two species differ from each other by the primordial hyphae near the pileus margin, which are one- or two-celled in *R*. *zephyrovelutipes* (average of all collections 1.3) and mainly two- to three-celled in *R*. *pseudopeckii* (average 2.4). Two further similar species with a trichodermal suprapellis are *R*. *rheubarbarina* differing by mostly unbranched subterminal cells of hyphal terminations in the pileipellis and *R*. *cynorhodon* with longer spores and more prominent spore ornamentation.

### Key to species of *Russula* subsect. *Roseinae* known for America

Names of species presented as new to science in the present publication are written with bold letters.

1A. Cap pale rosy, when dry radially cracking near margin, primordial hyphae usually one-celled, multiple (often more than three) narrow and short hyphal terminations originate from an abruptly inflated large cell of the subpellis                            *R*. *rimosa*
1B. Cap darker red or not radially cracking near the margin, primordial hyphae formed by mainly two or more cells, usually only one or two hyphal terminations are attached to inflated cells of subpellis                            2
2A. Pileus usually with umbo, cuticle shiny, viscid or glutinous, primordial hyphae with terminal cells in average longer than 25 μm, apically narrowed and often with lateral projections or nodes, strictly associated with conifers                            *R*. *peckii*
2B. Pileus often without umbo and not with a shiny cuticle, dry or slightly viscid, primordial hyphae with terminal cells shorter than 25 μm, apically obtuse, not strictly associated with conifers                            3
3A. Suprapellis near the pileus center being an epithelium composed of inflated elements with terminal cells mainly wider than 4 μm                            4
3B. Suprapellis near the pileus center being a trichoderm or transitioning to an epithelium, terminal cells mainly cylindrical and narrower than 4 μm                            7
4A. Primordial hyphae composed mainly of one or two, equally wide cells originating from inflated cells of the subpellis, terminal cells of hyphae near the pileus center not lobate *R*. *rubellipes*
4B. Primordial hyphae of mainly of 2–4 cells, terminal cells of hyphae near the pileus center lobate or not lobate                            5
5A. Guaiac reaction negative on both stipe and gills *R*. cf. *minutula* s. Looney et al. 2021 [20]
5B. Guaiac reaction negative on stipe, but positive on gills                            6
6A. Terminal cells of hyphae near the pileus center often lobate                            *R*. *cordata*
6B. Terminal cells of hyphae near the pileus center not lobate, snowman-shaped hyphal terminations near the pileus center present                            ***R*. *oreomunneae***
7A. Guaiac reaction negative on both stipe and gills                            8
7B. Guaiac reaction negative on stipe, but positive on gills                            10
8A. Average spore length < 7 μm, subterminal cells of hyphal terminations in pileipellis mainly unbranched                            *R*. *rheubarbarina*
8B. Average spore length >7 μm, subterminal cells mainly branched                            9
9A. Pleurocystidia thick-walled, terminal cells of hyphae near the pileus margin long and narrow
(Q-value: 4–5), width of terminal cells of primordial hyphae near pileus margin ≥ 3 μm *R*. *cardinalis*
9B. Pleurocystidia thin-walled, terminal cells of hyphae near the pileus margin short and stout
(Q-value 2.5–3.5), width of terminal cells of primordial hyphae near pileus margin ≤ 3 μm
                            ***R*. *cornicolor***
10A. Average spore length > 7 μm and average height of ornamentation > 1 μm
                            ***R*. *cynorhodon***
10B. Average spore length < 7 μm and average height of ornamentation < 1 μm                    11
11A. Subterminal cells of hyphae in pileipellis predominantly unbranched                *R*. *rheubarbarina*
11B. Subterminal cells of hyphae in pileipellis predominantly branched                            12
12A. Primordial hyphae near pileus margin 1–2 celled (av. 1.3) and subterminal cells of primordial hyphae frequently branched                     ***R*. *zephyrovelutipes***
12B. Primordial hyphae near pileus margin 2–3 celled (av. 2.4) and subterminal cells of primordial hyphae unbranched                            *R*. *pseudopeckii*

## Discussion

This study of Panamanian species belonging to *Russula* subsection *Roseinae* was facilitated by the fact that North American species of the subsection are well known. For the majority of the taxa described in this subsection, sequence data are available [15]. *Russula rimosa*, for which molecular sequence analyses were unsuccessful, has been morphologically redescribed in detail by Adamčík and Buyck [18] using type material. Furthermore, our search of Genbank sequences could not detect any Latin American *Russula* species that occurs outside the American continents. In the present study, we checked all the descriptions of *Russula* species reported from South and Central America and concluded that are no further described species that match the morphological concept of species in the subsection *Roseinae*. Similar to species of subsect. *Roseinae* is *Russula humboldtii* Sing. a species with red pileus, mild taste, cream coloured spore print and primordial hyphae described from oak forests of Columbia [42]. However, this species is probably not a member of subsect. *Roseinae* and differs clearly from all species described here by larger spores (9–11 × 7.5–9.8 μm), long septate primordial hyphae and a stipe that is staining brownish in age. The subsection *Roseinae* represents a monophyletic lineage which is characterized by a distinctive set of morphological characteristics. The eosin red reaction of the context to sulfo vanillin can still be recognized in old herbarium specimens and is therefore efficiently facilitating the assignment of old specimens to the subsection [20]. However, we realised during the fieldwork for this study, that there are also some Panamanian species with an eosin red colour reaction that do not belong to subsect. *Roseinae*.

In other lineages of the genus, it is much more complicated to identify undescribed species from Latin America, because recent molecular studies are lacking, the taxonomic concept of species described only by morphology is unclear and because distribution areas and ecological amplitudes of *Russula* species in the tropics are unknown. Up to now, 77 *Russula* species are described from the region [12]. A larger part of these taxa is unlikely to occur in tropical montane forests of the Chiriquí region in Western Panama though, because they are either limited to lowland tropical habitats or associated with *Nothofagus* spp. in temperate regions of South America. Some species can be excluded because they form sequestrate fruiting bodies, which is so far not recorded for Panamanian Russulaceae.

An unambiguous assignment of recent specimens to taxa that have been described a long time ago is hardly possible without detailed investigation of type material. In many cases it is difficult or impossible to retrieve DNA of sufficient quality for molecular identification, so that detailed microscopical re-descriptions are necessary. Additionally, it is often difficult to loan type material from abroad and even if detailed morphological data of the type collection is available, the assignment of recent collections can still be difficult due to the morphological variability of many species that is not covered by existing descriptions [43]. If the type material is non-existent or in a bad condition, it is necessary to find collections that a) match the morphological concept of the original description b) were collected in an area geographically close to the type locality and c) were collected in a similar habitat as the holotype. Ideally, a neo- or epitype is designated, allowing a molecular characterization and an unambiguous classification of the taxon [44].

Some *Russula* species reported from Panama have been described a long time ago and the correct identification is doubtful in some cases. The specimen identified as *R*. *mexicana* by Hennicke and Piepenbring [11], for example, represents one of the newly described species from subsect. *Roseinae* according our investigation of the corresponding collection.

This reduces the number of known Russula species from Panama to eight and adding the four new species described in this study leads to a total number of twelve species of *Russula* known from Panama. Taking into consideration that up to now only about 8% of the estimated 50.000 fungal species from Panama are documented [8, 9], the expected number of *Russula* species existing in Panama should be approximately 150. A study including metabarcoding of root tips in a small area of forest in the Fortuna area in Northwestern Panama dominated by *Oreomunnea* trees revealed a dominance of *Russula* spp. among ECM mycobiota with 40 OTUs being detected [45]. These results suggest that the actual diversity of *Russula* species in tropical montane forests in Panama is much higher than the currently twelve reported species.

Sequences retrieved from root samples from the sampling site in the Fortuna area prove that *R*. *cornicolor* and *R*. *oreomunneae* are associated with the ectomycorrhizal tree species *Oreomunnea mexicana* [13]. An additional association with *Quercus* species is possible, because oaks are present in some sampling sites of both species. Furthermore, *Quercus* species and *O*. *mexicana* frequently co-occur in Panama and a study by Smith et al. [46] detected a high overlap between ectomycorrhizal communities of co-occuring trees. For *Russula zephyrovelutipes* and *Russula cynorhodon*, *Quercus* species were the only ectomycorrhizal trees present at all sampling sites, which are located in the region of the Barú volcano.

Currently, about 15 percent of the known vascular plant species reported for Panama are considered to be endemic [47]. An oak species composition analysis recovered nine areas of montane oak endemism in the region between Panama and Mexico that are separated by valleys with a climate that is unsuitable for oaks [48]. Monitoring projects in neighbouring countries and regions are necessary, to determine if the four newly described species represent local endemic species of Western Panamá or endemic species of neotropical montane *Quercus*-forests.

The currently available data suggests that the ancestors of the four newly described species from subsection *Roseinae* migrated several times into the area, because the new species are not closely related to each other but are placed in distinct lineages with closest relatives known from Asia or North America. This study is the first to report *Roseinae* in Mesoamerica and there are no records of any member of the subsection from any region between the eastern USA and western Panama, although tropical montane forests with *Quercus* species are present in several parts of the area [49]. The records in this study represent the most southern records of species of subsection *Roseinae* in America and are the first proof of their presence in the neotropics. Exploring the species diversity of *Roseinae* in other geographic regions with suitable habitats, especially in Central America and Central Asia are necessary for a better understanding of the complex migration patterns during the evolution of the lineage.

## Supporting information

S1 Table Micromorphological comparison of the four newly described species from Chiriquí, Panama. Measurements for each collection are listed individually. TC = terminal cells of hyphae in pileipellis. Values are averages of 30 measurements for spores and TC and averages of 20 measurements for all other characters. Distinguishing characters are in dark grey shaded boxes and distinguishing values are in bold.
(XLS)

S2 Table Public database sequences. Sequences retrieved from public databases used in the phylogenetic analysis.
(XLSX)
